# Sharing genomic data from clinical testing with researchers: public survey of expectations of clinical genomic data management in Queensland, Australia

**DOI:** 10.1186/s12910-020-00563-6

**Published:** 2020-11-19

**Authors:** Miranda E. Vidgen, Sid Kaladharan, Eva Malacova, Cameron Hurst, Nicola Waddell

**Affiliations:** 1 QIMR Berghofer Medical Research Institute, 300 Herston Road, Herston, QLD 4006 Australia; 2 School of Public Health, Curtin University, Perth, WA 6102 Australia; 3 School of Population and Global Health, UWA, Perth, WA 6009 Australia

**Keywords:** Genomic, Public views, Health information, Data sharing, Secondary use, Data linkage

## Abstract

**Background:**

There has been considerable investment and strategic planning to introduce genomic testing into Australia’s public health system. As more patients’ genomic data is being held by the public health system, there will be increased requests from researchers to access this data. It is important that public policy reflects public expectations for how genomic data that is generated from clinical tests is used. To inform public policy and discussions around genomic data sharing, we sought public opinions on using genomic data contained in medical records for research purposes in the Australian state of Queensland.

**Methods:**

A total of 1494 participants completed an online questionnaire between February and May 2019. Participants were adults living in Australia. The questionnaire explored participant preferences for sharing genomic data or biological samples with researchers, and concerns about genomic data sharing.

**Results:**

Most participants wanted to be given the choice to have their genomic data from medical records used in research. Their expectations on whether and how often they needed to be approached for permission on using their genomic data, depended on whether the data was identifiable or anonymous. Their willingness to sharing data for research purposes depended on the type of information being shared, what type of research would be undertaken and who would be doing the research. Participants were most concerned with genomics data sharing that could lead to discrimination (insurance and employment), data being used for marketing, data security, or commercial use.

**Conclusions:**

Most participants were willing to share their genomic data from medical records with researchers, as long as permission for use was sought. However, the existing policies related to this process in Queensland do not reflect participant expectations for how this is achieved, particularly with anonymous genomics data. This inconsistency may be addressed by process changes, such as inclusion of research in addition to clinical consent or general research data consent programs.

## Background

Australia is in the midst of a surge in public investment in clinical genomics through programs that focus on accelerated health service implementation and funding for translational research [1]. In 2016, the Queensland State government committed $25 million to the accelerated implementation of genomics into public healthcare [2]. Queensland has a population of approximately 5 million and a statewide public health system. The mainstreaming of genomics into Queensland’s clinical practice will result in an increasing amount of genomic data being held by the health system.

Australia’s public health system is predicated on patient health data, in both aggregated and individual forms, being used for research and quality improvement activities to enhance patient services and outcomes [3]. In Queensland, there is legislation and associated policies that outline how and when health information can be used for research [4–7]. There are currently no specific policies related to sharing of clinically derived genomic data with researchers. Any application to access genomic data for research purposes is considered under the policies related to general health information [4, 5] and by Human Research Ethics Committees (HREC) using national standards [8].

In Queensland, anonymised health information can be shared with researchers for ethically approved projects without individual consent in circumstances where health information cannot be directly or indirectly linked back to the patient [9]. Under certain circumstances, identifiable or re-identifiable health information can also be shared with researchers for ethically approved research [4]. This can occur when: (a) the patient has provided specific consent to participate in the project, or (b) the Director General of Health, or his/her delegate, has approved a Public Health Act (PHA) application in circumstances where researchers are unable to obtain individual consent, or it is deemed inappropriate or practically infeasible to contact patients. When a PHA application is sought, the research project must fulfil a waiver of consent criteria stipulated in the National Statement on Ethical Conduct in Human Research [8].

There has been a number of international studies that looked at public, patient and research participant perspectives on various aspects of genomic data sharing. These studies inquired about trustworthiness, risk, sharing preferences and concerns [10–15]. Participant preference for sharing their genomic health information with researchers can differ due to jurisdictional, societal and demographic differences between study populations, and types of linkage to personal identifiers proposed for the study [10–15]. Recent work in Australia on health information and biospecimen sharing has observed an overall public willingness to participate in research [16–18]. This is caveated by differences of opinions in use of identifiable data [17, 18], the need to know who the data recipients are [16, 17], and a desire for autonomy in providing permission [16, 18].

This study explores public opinions related to the sharing of genomic data for research from clinical records in the context of current policies. Questions were framed around preferences relating to identifiable and anonymous genomic data or biological samples. The questionnaire was drafted in the context of current Queensland Health (QH) policies around data sharing for research purposes.

## Methods

### Recruitment

Eligible participants were adults aged 18 years or over who were residents of Australia, according to the postcode of residence provided. Recruitment was via a self-selected sampling strategy of the general population that utilised electronic direct marketing, QIMR Berghofer’s magazine (LifeLab), and social media posts on QIMR Berghofer’s Facebook and Twitter accounts. The electronic direct marketing contacted individuals who were on a QIMR Berghofer mailing list and who have previously subscribed to be notified about the Institute’s future research. The questionnaire was available online for 16 weeks from February 2019 to May 2019.

We surveyed both Queensland and non-Queensland residents. The participant groups from different states were included since: (1) there are cross jurisdictional agreements for the use of QH services by patients from different states, (2) residents from different states may have previously been residents of Queensland, and (3) some Queensland resident may not have experienced QH services. This questionnaire is based on a perception of QH and that perception can come from multiple sources that are not just based on personal experiences.

### Questionnaire design

The cross-sectional online questionnaire (Additional file 1) comprised three sections: (A) socio-demographics, (B) permissions and preferences for either genomic data or biological samples sharing, and (C) concerns about genomic data sharing. The questionnaire contained 16 questions (Additional file 1: Section A: Q1–Q7, Section B: Q8–Q14, Section C: Q15–Q16) and took about 10–15 min to complete. Each section used a mixture of questions and answer formats including: single response, multiple responses, categorical responses, Likert scales, and open text box.

Questions related to permission for genomic data sharing were informed by the current QH policy on data sharing and consent (Q8–Q11) (Additional file 1). Questions related to preferences for genomic data sharing (Q12–Q14) are based on the categories and options used by the Australian Genomics Health Alliance CTRL platform for dynamic consent [19], which were derived from the Global Alliance for Genomic and Health’s Data Use Ontology technical standard [20]. Response options provided for concerns about genomic data sharing were based on a previous international public survey [21, 22], and then modified for this study’s purpose based on feedback about comprehension from consumer representatives and expert community members from the Queensland Genomics Community Advisory Group (n = 11).

Participants were supplied with a definition of the terms *identifiable* and *anonymous* in the questionnaire. Identifiable was defined as “your genomic data or biological sample and your personal information (i.e. your name, date of birth or contact details)” and anonymous was defined as “information will be limited to your genomic data or biological sample only. Your personal information would not be linked to your genomics data or sample (i.e. your name, date of birth or contact details)” (Additional file 1). Depending on the genomic data set requested by researchers, these categories and the provided definitions are a simplification of the complexity of anonymising genomic data in practice. The choice of definitions provided was based on the terms used in QH data sharing policies [9] and testing of comprehension by community members.

### Analysis

For comparison of groups, the response categories of some demographics were grouped into fewer categories. Age was collapsed from the 10-year increments into three categories: 18–34 years, 35–54 years, or 55 years and over. Education was collapsed to university or non-university educated, while state to Queensland or non-Queensland based on postcode. Participants who were ‘unsure’ if they have had genetic or genomic testing were combined with those who answered ‘no’ to this question. For the gender demographic question, participants who selected ‘other’ were excluded from gender-based analysis due to the low number (n = 8), but were included in all other analyses. We categorised postcodes into areas of most disadvantaged to most advantaged using Socio-Economic Indexes for Areas (SEIFA) Index of Relative Socio-Economic Advantage and Disadvantage (IRSAD) [23]. SEIFA local government postcode list was also used to categorise postcodes to metropolitan or regional categories.

All statistical inference was conducted using available case analysis (i.e. only pairwise-complete participants were included for the chi-square tests). Categorical data was summarised using counts and percentages and compared using a chi-squared test. Statistical significance was set at *p* ≤ 0.01. Questions with multiple response options were dichotomised to allow for groupwise comparisons (Additional file 2). The open text response question (Q16) related to concerns was thematically analysed using a manual process. Analyses were conducted in Stata (version 15.1).

## Results

### Demographics

A total of 1658 participants started the questionnaire. After exclusion of those who did not respond after demographic questions (n = 161), and those either with international postcodes or without an identifiable Australian postcode (n = 3), there were 1494 responses that qualified for the analysis.

Participants ranged from 18 to over 75 years of age (Table 1). The majority of them were aged 55 years or more (71.4%, n = 1066), and female (66.8%, n = 994), while participants aged 18–35 years being under represented (3.9%, n = 58). Participants were disproportionally from higher socioeconomic status areas (combined ISRAD 3–5: 77.6%, n = 1143). The majority of participants were university educated (61.5%, n = 918) and almost one-third have worked in healthcare (31.8%, n = 473) (Table 1). Over half of participants were Queensland-based (55.6%, n = 820) as this was the target audience.

**Table 1:** Socio-demographic characteristics of questionnaire participant

Demographic variables (N = 1494)	All participants N (%)	Australian population %
*Gender* (N = 1489, 99.7%)^a^		
Male	487 (32.7)	49
Female	994 (66.8)	50.7
Other	8 (0.5)	ND
*Age (years)* (N = 1493, 99.9%)		
18–34	58 (3.9)	31
35–54	369 (24.7)	36
55+	1066 (71.4)	37
*Education* (N = 1492, 99.9%)		
University	918 (61.5)	23
Non-university	574 (38.5)	77
Didn't complete year 10	21 (1.4)	11
Year 10 or equivalent	107 (7.2)	13
Year 12 or equivalent	155 (10.4)	19
TAFE/Apprenticeship or equivalent	291 (19.5)	30
*State of residence* (N = 1475, 98.7%)		
Queensland	820 (55.6)	20.1
Non-Queensland	655 (44.4)	79.9
New South Wales	249 (16.9)	32.0
Victoria	174 (11.8)	25.3
Western Australia	79 (5.4)	10.6
South Australia	61 (4.1)	7.2
Tasmania	35 (2.4)	2.2
Australia Capital Territory	49 (3.3)	1.7
Northern Territory	8 (0.5)	0.9
*Worked in life science* (N = 1487, 99.5%)		ND
No	1384 (93.1)	
Yes	103 (6.9)	
*Worked in healthcare* (N = 1487, 99.5%)		
No	1014 (68.2)	
Yes	473 (31.8)	12.6 ^b^
*Had genetic or genomic testing* (N = 1492, 99.9%)		ND
No	920 (61.7)	
Yes	435 (29.2)	
Unsure	137 (9.2)	
*SEIFA (ISRAD)* (N = 1474, 98.7%)		
1 (most disadvantages)	136 (9.2)	20
2	195 (13.2)	20
3	244 (16.6)	20
4	405 (27.5)	20
5 (most advantaged)	494 (33.5)	20
*Location* (N = 1474, 98.7%)		
Metropolitan	916 (62.1)	67
Regional	558 (37.9)	33

*ND* no data available from 2016 Census

^a^Total number of participants who responded to question and percentage of total questionnaire respondents

^b^Percentage of workforce age population working in health and social services sector at time of 2016 Census

One-third of participants reported that they have had a genetic or genomics test in the past (29.2%, n = 435), which could have been clinical diagnostic test, participation in genomic research, or direct-to-consumer testing (health or recreational testing as defined previously [24]).

### Permission for genomic data sharing

Overall, most agreed (86.2%, n = 1281) that QH should ask individual permission before sharing identifiable genomic data with researchers, but only one-third (35.7%, n = 530) considered individual permission necessary when sharing anonymous genomic data (Table 2).

**Table 2:** Participant preferences when seeking permission for sharing genomic data and biological samples with researchers

	Identifiable genomic data N (%)	Anonymous genomic data N (%)	Identifiable biological samples N (%)	Anonymous biological samples N (%)
*Should Queensland Health ask your permission before allowing researchers to access the following from your medical record?*				
Total number of participants who responded^a^	1487 (99.5)	1484 (99.3)	1484 (99.3)	1476 (98.8)
Strongly agree	858 (57.7)	260 (17.5)	820 (55.3)	256 (17.3)
Agree	423 (28.5)	270 (18.2)	432 (29.1)	265 (18.0)
Undecided	61 (4.1)	170 (11.5)	71 (4.8)	168 (11.4)
Disagree	101 (6.8)	490 (33.0)	107 (7.2)	486 (32.9)
Strongly disagree	44 (3.0)	294 (19.8)	54 (3.6)	301 (20.4)
Overall agreement for asking permission^b^	1281 (86.2)	530 (35.7)	1252 (84.4)	521 (35.3)
Overall disagreement for asking permission^c^	145 (9.8)	784 (52.8)	161 (10.9)	787 (53.3)
*How often should Queensland Health ask for permission to give researchers access to the following from your medical record?*				
Total number of participants who responded^a^	1486 (99.5)	1481 (99.1)	1481 (99.1)	1476 (98.8)
Every time	960 (64.6)	287 (19.4)	956 (64.6)	291 (19.7)
Sometimes	59 (4.0)	93 (6.3)	54 (3.7)	92 (6.2)
Only once	391 (26.3)	537 (36.3)	386 (26.1)	525 (35.6)
Never	76 (5.1)	564 (38.1)	85 (5.7)	568 (38.5)

^a^Percentage for level of agreement is given as total number of participants who responded

^b^Overall agreement is calculated as the sum of ‘agree’ and ‘strongly agree’

^c^Overall agreement is calculated as the sum of ‘disagree’ and ‘strongly disagree’

Two-thirds nominated that QH should ask their permission either every time or sometime before their identifiable genomics data (68.6%, n = 1019) would be shared with researchers, with a further quarter preferring to be asked only the first time (26.3%, n = 391) (Table 2). For anonymous genomics data, preferences for being asked only once was higher (36.3%, n = 537) than the need to ask at least sometimes (25.7%, n = 380). There was little difference in the observed preferences for seeking permission for biological samples when compared with genomic data (Table 2).

Under half of all participants stated they would allow another person to give permission on their behalf, once they are no longer able to (43.1%, n = 642), with family members being the most preferred option (61.1%, n = 392), followed by nominated legal representative (e.g. power of attorney) (45.8%, n = 294) (Table 3).

**Table 3:** Participant preferences when seeking permission for sharing genomics data when patient is no longer able

	Total N (%)
*Do you think someone else should be able to give permission for researchers to access your anonymous genomic data from medical records if you are no longer able? (N* = *1491, 99.80%)*	
No, only I can give permission	353 (23.7)
No, data freely available	496 (33.3)
Yes	642 (43.1)
*If Yes, who would you prefer to give permission for your anonymous genomic data to be used in research on your behalf? (you can select multiple answers) (N* = *642)*	
Family member	392 (61.1)
Nominated legal respresentative	294 (45.8)
Doctor	125 (19.5)
Human Research Ethics Committee (HREC)	121 (18.9)
Data governance	61 (9.5)

### Preferences for genomic data sharing

There were substantial variations in participant preferences for the organisations with which they would share their genomic data, ranging from 13.0 to 91.9% for anonymous and from 0.9 to 72.6% for identifiable data (Table 4). Overall, participants were between 12.1 and 31.1% less likely to share their identifiable than anonymous genomic data.

**Table 4:** Participant preferences for organisation that they would choose to share their genomics data

	Total number of participants who responded (%)	Of the participants who responded			
		Yes N (%)	Percentage difference (%)^a^	No N (%)	Unsure N (%)
*What organisations would you share your anonymous genomic data with?*					
Australian universities and research institutes	1481 (99.1)	1361 (91.9)	–	49 (3.3)	71 (4.8)
Australian not-for-profit research organisations	1480 (99.1)	1359 (91.8)	–	50 (3.4)	71 (4.8)
Australian government	1453 (97.3)	720 (49.6)	–	498 (34.3)	235 (16.2)
Overseas universities and research institutes	1462 (97.9)	797 (54.5)	–	432 (29.6)	233 (15.9)
Overseas not-for-profit research organisations	1465 (98.1)	842 (57.5)	–	386 (26.4)	237 (16.2)
Overseas governments	1449 (97.0)	247 (17.1)	–	1016 (70.1)	186 (12.8)
Commercial company	1448 (96.9)	247 (17.1)	–	981 (67.8)	220 (15.2)
Publically available	1443 (96.6)	187 (13.0)	–	1094 (75.8)	162 (11.2)
*What organisations would you share your identifiable genomic data with?*					
Australian not-for-profit research organisations	1482 (99.2)	1076 (72.6)	− 19.3	189 (12.8)	217 (14.6)
Australian universities and research institutes	1484 (99.3)	1062 (71.6)	− 20.3	188 (12.7)	234 (15.8)
Australian government	1453 (97.3)	268 (18.4)	− 31.1	766 (52.7)	419 (28.8)
Overseas not-for-profit research organisations	1458 (97.6)	341 (23.4)	− 31.1	701 (48.1)	416 (28.5)
Overseas universities and research institutes	1462 (97.9)	402 (27.5)	− 30.0	636 (43.5)	424 (29.0)
Overseas governments	1450 (97.1)	36 (2.5)	− 14.6	1241 (85.6)	173 (11.9)
Commercial company	1449 (97.0)	43 (3.0)	− 14.1	1209 (83.4)	197 (13.6)
Publically available	1450 (97.1)	13 (0.9)	− 12.1	1324 (91.3)	113 (7.8)
*What types of research would you share your anonymous genomic data with?*					
Research specific to a condition I have	1480 (99.1)	1407 (95.1)	–	30 (2.0)	43 (2.9)
Research into other diseases and conditions	1481 (99.1)	1341 (90.6)	–	46 (3.1)	94 (6.4)
General population health research	1483 (99.3)	1285 (86.7)	–	95 (6.4)	103 (7.0)
Ancestry research	1467 (98.2)	961 (65.5)	–	296 (20.2)	210 (14.3)
Unspecified future research	1467 (98.2)	717 (48.9)	–	322 (22.0)	428 (29.2)

^a^Difference between participants that would share their anonymous and their identifiable genomic data

The majority of participants would share their genomic data with Australian not-for-profit organisations (Anonymous: 91.9%, n = 1361; Identifiable: 72.6%, n = 1076), or universities and research institutes (Anonymous: 91.8%, n = 1359; Identifiable: 71.6%, n = 1062) (Table 4).

Overseas governments (Anonymous: 17.1%, n = 247; Identifiable: 2.5%, n = 36), commercial companies (Anonymous: 17.1%, n = 247; Identifiable: 3.0%, n = 43) and data being made publically available (Anonymous: 13.0%, n = 187; Identifiable: 0.9%, n = 13) rated the lowest for sharing both anonymous and identifiable genomic data (Table 4).

Nearly all participants would agree to share anonymous genomic data for research of a condition they have (95.1%, n = 1407), other diseases or conditions (90.6%, n = 1341), or general public health research (86.7%, n = 1285). About half of all participants would share their anonymous genomic data for unspecified future research (48.9%, n = 717) (Table 4).

### Concerns about genomic data sharing

The majority of participants expressed high concerns about potential insurance discrimination (83.8%, n = 1218), marketing companies (83.2%, n = 1211), employment based discrimination (80.7%, n = 1174), genomic data being made publically available (70.4%, n = 1026), stigmatisation (64.1%, n = 932), and ethnic/racial discrimination (63.0%, n = 912) (Table 5). In comparision, under one-fifth of participants felt the same high concern about family finding out about their health results (19.8%, n = 288), upsetting genetic relatives (19.9%, n = 290), or data being used for quality improvement in QH diagnostics (17.9%, n = 260).

**Table 5:** Participants levels of concern associated with sharing genomic data for health records with researchers

	Total number of participants who responded N (%)	Of the participants who responded				
		Very concerned N (%)	Moderately concerned N (%)	Somewhat concerned N (%)	Slightly concerned N (%)	Not concerned N (%)
Insurance companies using my genomic data to discriminate against me	1454 (97.3)	1218 (83.8)	89 (6.1)	61 (4.2)	39 (2.7)	47 (3.2)
Marketing companies targeting me to sell me products	1455 (97.4)	1211 (83.2)	112 (7.7)	66 (4.5)	32 (2.2)	34 (2.3)
Employers using my genomic data to discriminate against me	1455 (97.4)	1174 (80.7)	92 (6.3)	65 (4.5)	29 (2.0)	95 (6.5)
My genomic data being made publicly available	1457 (97.5)	1026 (70.4)	153 (10.5)	118 (8.1)	86 (5.9)	74 (5.1)
Being labelled or stigmatised in some way	1454 (97.3)	932 (64.1)	136 (9.4)	114 (7.8)	84 (5.8)	188 (12.9)
Ethnic or racial discrimination	1447 (96.9)	912 (63.0)	107 (7.4)	97 (6.7)	62 (4.3)	269 (18.6)
Privacy of my personal details (e.g. name, date of birth, address)	1457 (97.5)	875 (60.0)	234 (16.1)	152 (10.4)	109 (7.5)	87 (6.0)
My genomic data being used for research without my permission	1455 (97.4)	684 (47.0)	259 (17.8)	174 (12.0)	147 (10.1)	191 (13.1)
Police using genomic databases with my details to investigate crimes	1453 (97.3)	554 (38.1)	162 (11.2)	167 (11.5)	161 (11.1)	409 (28.2)
Receiving information about my future health that has no treatment option	1455 (97.4)	521 (35.8)	232 (16.0)	224 (15.4)	174 (12.0)	304 (20.9)
My family finding out about my health results	1453 (97.3)	288 (19.8)	172 (11.8)	163 (11.2)	175 (12.0)	655 (45.1)
Upsetting my genetic relatives, because my genomic information is similar to theirs	1454 (97.3)	290 (19.9)	166 (11.4)	171 (11.8)	222 (15.3)	605 (41.6)
My genomic data being used by Queensland Health to improve services or diagnostic tests	1452 (97.2)	260 (17.9)	243 (16.7)	216 (14.9)	193 (13.3)	540 (37.2)

The most common themes in the open text responses to concerns (n = 247) related to data security (22.3%, n = 55), commercial use or gains (13.4%, n = 33), autonomy in choosing to participate (10.9%, n = 27), and the use of genomic data without consent (10.1%, n = 25) (Table 6). Although the open text question intended to identify any other concerns about sharing genomic data from medical records for research, about 14.2% of respondants conveyed their support for sharing data for research purposes.

**Table 6:** Summary of identified themes from open text box responses of participant concerns

Themes of concerns (N = 247)	N (%)^a^
Data security	55 (22.3)
Commercial use or gains	33 (13.4)
Autonomy of choice	27 (10.9)
Consent	25 (10.1)
Implications for self	20 (8.1)
Personal ethics on research type	18 (7.3)
Privacy	16 (6.5)
Access	12 (4.9)
Trust	11 (4.5)
Misuse	10 (4.1)
Family implications	9 (3.6)
Data management	7 (2.8)
Interest in area of research	5 (2.0)
Questionnaire comment	5 (2.0)
Objection to sharing	4 (1.6)
Positive response to sharing	35 (14.2)

^a^326 identified themes of concern identified from free-text comments of 247 respondents. Percentage calculated from number of participants that provided a text box response to Q14 (N = 247)

### Comparing data sharing preferences across groups

Analyses based on demographic participant groups revealed some differences across the groups (Additional file 2). Participants who were < 55 years old, had worked in health care, or were university-educated indicated that the permission needed to be sought for the use of identifiable genomics data and this should occur more than once (*p* ≤ 0.01) (Additional file 2: Tables S1 and S2). Participants who did not reside in Queensland and were female indicated that permission should be required more than once for both identifiable genomic data and biological samples (*p* ≤ 0.01).

Participants who had previous experience with genetic testing, were ≥ 55 years or under 35 years old, or from Queensland more often agreed to sharing their anonymous genomic data for ancestry research (all *p* ≤ 0.01) (Additional file 2: Table S6). Conversely, participants who have worked in health care, in life sciences, or have attained university education less often agreeed to sharing genomics data for ancestry research or unspecified future research (*p* ≤ 0.01) (Additional file 2: Table S6). Preference for third party permission for use of anonymous genomics data varied depending on age, ISRAD at place of residence, education, and experience working in life science and with genetic testing (Additional file 2: Table S3). Participants preference for organisations that they would choose to share anonymous and identifiable genomics data varied between each of the demographic variables (*p* ≤ 0.01) (Additional file 2: Table S4 and S5).

A smaller proportion of Queensland residents and those with experience of genetic testing expressed high or moderate concern about sharing their genomic data compared to non-Queensland residents and those with no experience of genetic testing (Additional file 2: Table S7a and S7b).

## Discussion

Our questionnaire of public opinions of sharing genomic data from medical records found that participants were willing to share their genomic data with researchers. However, our results suggest that this willingness is predicated on several caveats related to; availability of personal details, organisation that will be the recipient, and type of research being undertaken. These results reflect the findings of previous studies related to health information and genomic data that have been observed in other public [16] and patients studies in Australia [17, 18]. Concerns about types of research related to topics with a higher level of ethical consideration, for example embryos and stem cells [8]. Conversely, participants had high levels of support for using their genomic data in research of diseases they had, other diseases, or general population health. An important consideration is that unless ‘extended’ consent (as defined by National Statement [8]) for research into other disease and general population is collected in the clinical setting, there may be limitations in the ability to use anonymised genomics data for these research types without gaining individual consent. This is influenced by the National Statement [8] and HRECs applications of standards rather than QH policies.

In clinical genetic testing, there has been a debate surrounding the concept of genetic exceptionalism—which proposes that genetic and genomic data have special risks not observed in other types of health information and, therefore, needs different considerations in data management and patient consent [25]. Participants in this study did not indicate genetic exceptionalism views, with similar preferences for data sharing for both biological samples and genomics data. Participants in a global study (that included Australians) who viewed genomic data as exceptional, were more willing to participate in research than those without genomic exceptionalism views [22], thus indicating that even if QH took a genomic exceptionalism position and developed genomic data specific policies, people would still participate in research. Perhaps this suggests that if any genomic data specific policy changes occur, they should occur in tandem with patient consent mechanisms for research.

Participant expectations for sharing and permissions are fundamentally different between anonymised and identifiable genomic data. This difference is reflected in health information sharing polices, but not to the extent expected by the study participants. The health information sharing policies of QH [4] largely reflect the opinions of the majority of participants when considered in the context of identifiable genomic data. In that, permission needs to be sought before using identifiable genomic data for research purposes. However, participants who want permission sought from a third party preferred family or legally nominated representatives to give consent when they are no longer able to consent, rather than data governance or HREC that are used in current policies.

In contrast, QH’s current policies around sharing of anonymous health information [9] do not reflect participant’s expectations in relation to genomic data. While one-third of participants would accept for their data to be used without permission for research uses, the majority would require permission to be sought at least once. There has been work within Australia to create a nationally consistent clinical genomic consent form that includes an option for participation in anonymised research [26]. Implementation of research consent options into clinical consent forms could address this discrepancy between participant preferences and policy observed in this study.

In our analysis, age, educational, and work related factors were most commonly associated with differences in participant preferences for genomic data sharing. Specifically, those who were < 55 years old, university educated or worked in healthcare tended towards a reluctance to share their genomic data. Age, experience with poor health or genetic testing, and educational attainment have been identified as factors in both international and Australian studies of people’s willingness to share health information and genomic data with researchers or to participate in biobanking [10–12, 14, 16, 18, 22]. The association with age and educational attainment tend to vary depending on the location of survey participants [10–12, 16], whilst experience with genomics or poor health is consistently associated with a willingness to share with research [14, 18, 22]. In this study, experience of genetic testing did not influence preferences on whether and how often researchers need to seek permission for the use of genomic data (Additional file 1: S1 and S2), but these participants had less concern associated with sharing genomic data (Additional file 1: S7a and S7b). Residents of Queensland were less concerned with sharing of genomics data than respondents from other states, which reflects previous findings [16]. Other studies have identified ethnicity/race and religiosity as influencing willingness to participate in genomic research [12, 27], which were not considered in this study.

The present study demonstrated apprehension for commercial organisations access genomic data for research that could be used for profit. This mistrust of for-profit companies has similarly been observed in studies based in other countries [10, 14]. Notably, participants stated they were more likely to give permission to have overseas research organisations (not-for-profit, medical institutes and universities) have access to their genomic data than the Australian government. This is in direct contrast to a study where research by domestic governments was shown to be preferred over international researchers [28].

Implications for insurance and employment were of particular concern for participants. Australia does not have specific genetic discrimination laws. Anti-discrimination legislations at both federal and state level are used for genetic discrimination cases [29, 30]. In Australia, individual health information is not used to determine private health insurance premiums [31]. For life insurance, companies are limited in what genetic information a policy applicant is required to disclose. The limiting of disclosure requirements for life insurance has come about through a moratorium [32] rather than through legislation, and it is likely to change over time.

Based on open text responses to concerns, the desire to be asked permission seemed to stem from participants seeking autonomy over the ability to participate in research, wishing for their participation in research to reflect personal priorities and ethics, and a desire to know about the research in which they would be participating. Concerns over genomic data use outside of intended research did not limit participant’s willingness to participate in genomic research, which confirms the findings of other studies [10, 22]. Interestingly, some study participants conveyed a positive response to sharing data for research inspite of their concerns. Further exploration of the barriers and motivators to research participation would be useful to inform QH policies on clinical data sharing with research.

### Policy directions and future work

The study highlights that current data sharing policies only partially reflect participant expectation on clinical genomic data sharing for research purposes. QH should explore policy or practice changes options, preferably with public consultation. This could be achieved through consultation with community, patient advocacy and advisory groups, which already exist within Queensland, or further research activities that engage specific groups. Surveys like this one set the scene for discussions, but are limited in granular details on what is a very complex topic.

There are groups within the population that are under-represented in this study and likely to require further engagement. In particular, the young (18–34 years), males, those with year 12 education or lower, and those living in lower socioeconomic areas. There are also populations that will need further consideration and engagement; patient groups, Aboriginal and Torres Strait Islanders communities, and cultural and linguistically diverse communities. Based on other work, these groups are likely to have different perspectives and expectations on sharing of clinical genomic data and biological samples for research purposes [11, 17, 33, 34].

To reflect participant expectations for genomic data management, not all changes need to be based on legislation and policy. They could also be addressed by documentation processes and programs. This study demonstrated that participants expect to be asked to share anonymous genomic data at least once. Mechanisms for ‘extended’ or ‘unspecified’ consent (as defined in National Statement [8]) could be included in clinical consent forms to provide an opt-in or opt-out option. A program within Australia has been working to standardise clinical consent for genomics, with clinical consent forms that include a research participation option [26]. However, in the development of these clinical consent forms, there were opposing views about seeking research consent in clinical settings. Gaining consent to research from patients outside of a clinical setting or establishing ways for patients to have ongoing control of consent, such as dynamic consent platforms, could alleviate these concerns. In Queensland, there was a trial of gaining broad consent to research from patients outside of clinical consultations in the Giving InFormation To Research (GIFTR) program [35]. Statewide application of a program like this may assist not only genomic research but other types of research as well. The options given will require ongoing investment, adjustment to consent wording in response to ethics guidance changes or legislative changes, and/or establishment of searchable information technology infrastructure to ensure current consent preferences can be applied.

### Limitations

The main limitation in the current study was there was a potential for bias due to over or under sampling of certain sub-populations of participants. Participants were predominantly females over 55 years in age and highly educated. This demographic spread is not representative of the general population [36]. While a larger number of participants did enable us to consider responses of sub-populations, there is still the potential for bias due recruitment strategy and self-selection. These included the questionnaire only being available online, and recruitment material being primarily directed towards people with previous engagement with a research organisation. Both of which may lead to a biased sample of participants that is not representative of the general population. Based on the findings of other research [18, 22], we anticipated that a questionnaire of a patient group may produce different views on genomics data sharing for research purposes. Investigation of this group’s perspective is an essential next step in the genomic data sharing discussion.

Through the open text box questions some participants reported feeling concerns as questions (Q15–16) were ambiguous, as participants were not directed to consider an *anonymous* or *identifiable* scenario. As such, the findings related to concerns should be considered with caution. Genomics and its associated issues are technically difficult and multifaceted to explain, as such they do not lend themselves well to a multiple-choice based questionnaires as it is hard to convey nuanced opinions [37]. In our questionnaire, participants were not given a definition of genomic data; therefore, responses were based on participant pre-existing understanding of genomic data, which we expect to vary greatly within the participants.

## Conclusions

In the coming years, QH will be a repository for a large amount of clinically derived genomic data. With this, QH is likely to receive more requests from researchers to access this data using the existing data access practises and policies. This study indicates that, especially in the context of anonymous data, current policies may not meet participant expectation for autonomy in choosing how their clinical genomic data is shared with researchers. Further consultation is needed with demographic groups, including those that were under-represented in this study, patients, and populations with special cultural considerations, including Aboriginal and Torres Strait Islander communities, and culturally and logistically diverse communities.

This study demonstrates a high degree of variability in participants’ willingness and preferences for sharing their genomic data with researchers. Some health information management policies do not fulfil the expectation of participants for genomics data sharing. Here we propose process-driven changes, such as research consent during or after clinical consultation of anonymous genomic data use, as a way of better representing expectations for permission for data sharing. What is most important is that there is an active decision on genomic data management rather than a continuation of existing data sharing policies without review. This will assist in aligning public expectations with health policy directives, whilst also including global genomic policy developments, bioethics considerations, and technology advancements.

## Supplementary information

**Additional file 1.** Questionnaire.

**Additional file 2.** Summary tables for statistical analysis of demographic groups.

## Data Availability

The datasets generated and analysed during the current study are not publicly available because participants did not give consent to share the data beyond the research team.

## Abbreviations

GIFTR: Giving InFormation To Research
HREC: Human Research Ethics Committee
IRSAD: Index of Relative Socio-Economic Advantage and Disadvantage
PHA: Public Health Act
QGHA: Queensland Genomics Health Alliance
QH: Queensland Health
SEIFA: Socio-Economic Indexes for Areas
