# Supplementary material for: Sharing genomic data from clinical testing with researchers: public survey of expectations of clinical genomic data management in Queensland, Australia
Source: BMC Med Ethics. 2020 Nov 19;21:119. doi: 10.1186/s12910-020-00563-6 (PMC7678081; doi:10.1186/s12910-020-00563-6)
Supplement: Supplementary file 1 — Additional file 1. Questionnaire. [file 12910_2020_563_MOESM1_ESM.docx]

**Supplementary Materials 1: Questionnaire**

**SECTION A. Demographic questions**

**Q1** Have you ever had genetic or genomics testing? (*examples: diagnostic test by a doctor, ancestry testing by a commercial company or genomic research*)

**Options:** Yes, No and Unsure

**Q2** Have you worked in genomics and/or life sciences?

**Options:** Yes and No

**Q3** Have you worked in health care?

**Options:** Yes and No

**Q4** What is your age?

**Options:** 18 – 24 years old, 25 - 34 years old, 35 – 44 years old, 45 – 54 years old, 55 – 64 years old, 65 – 74 years old, and 75 years or older

**Q5** What is your gender?

**Options:** Female, Male and Other

**Q6** What postcode do you live in?

**Options:** [text box]

**Q7** What is the highest level of education you have completed?

**Options:** Didn’t complete school to year 10, Year 10 / equivalent, Year 12 / equivalent, TAFE or trade qualification / apprenticeship, and University qualification

**SECTION B. Permission and preferences**

Your genomic data and biological samples (e.g. blood or tissue) from medical tests can be stored by Queensland Health as part of your medical records. Your medical record contains information about your medical history, test results and your contact details.

In the following questions we will refer to ‘**identifiable**’ or ‘**anonymous**’.

By *‘****identifiable****’* we mean your genomic data or biological sample **and** your personal information (i.e. your name, date of birth or contact details).

By *‘****anonymous****’* we mean information will be limited to your genomic data or biological sample only. Your personal information would not be linked to your genomics data or sample (i.e. your name, date of birth or contact details).

**Q8** Should Queensland Health ask your permission before allowing researchers to access the following from your medical record?

|  | Strongly agree | Agree | Undecided | Disagree | Strongly disagree |
| --- | --- | --- | --- | --- | --- |
| Identifiable genomic data | 🖵 | 🖵 | 🖵 | 🖵 | 🖵 |
| Anonymous genomic data | 🖵 | 🖵 | 🖵 | 🖵 | 🖵 |
| Identifiable biological samples | 🖵 | 🖵 | 🖵 | 🖵 | 🖵 |
| Anonymous biological samples | 🖵 | 🖵 | 🖵 | 🖵 | 🖵 |

**Q9** How often should Queensland Health ask for permission to give researchers access to the following from your medical record?

|  | Every time | Sometimes | Only once | Never |
| --- | --- | --- | --- | --- |
| Identifiable genomic data | 🖵 | 🖵 | 🖵 | 🖵 |
| Anonymous genomic data | 🖵 | 🖵 | 🖵 | 🖵 |
| Identifiable biological samples | 🖵 | 🖵 | 🖵 | 🖵 |
| Anonymous biological samples | 🖵 | 🖵 | 🖵 | 🖵 |

**Q10** Do you think someone else should be able to give permission for researchers to access your **anonymous** genomic data from medical records if you are no longer able?

**Options:** Yes (go to Q11), No, only I should be able to give permission (go to Q12), and No, my anonymous genetic data should be available to any researcher (go to Q12)

**Q11** Who would you prefer to give permission for your **anonymous** genomic data to be used in research on your behalf? *(you can select multiple answers)*

**Options:** Family member/ Next of kin, Person you legally nominate (e.g. power of attorney), Your doctor, Human Research Ethics Committee, and Queensland Health data governance

**Q12** What organisations would you share your **identifiable** genomic data with?

|  | Yes | No | Unsure |
| --- | --- | --- | --- |
| Australian not-for-profit research organisations (eg Cancer Council) | 🖵 | 🖵 | 🖵 |
| Australian universities and research institutes | 🖵 | 🖵 | 🖵 |
| Australian government | 🖵 | 🖵 | 🖵 |
| Overseas not-for-profit research organisations | 🖵 | 🖵 | 🖵 |
| Overseas universities and research institutes | 🖵 | 🖵 | 🖵 |
| Overseas governments | 🖵 | 🖵 | 🖵 |
| Commercial company | 🖵 | 🖵 | 🖵 |
| Anyone (Publicly available) | 🖵 | 🖵 | 🖵 |

**Q13** What organisations would you share your **anonymous** genomic data with?

|  | Yes | No | Unsure |
| --- | --- | --- | --- |
| Australian not-for-profit research organisations (eg Cancer Council) | 🖵 | 🖵 | 🖵 |
| Australian universities and research institutes | 🖵 | 🖵 | 🖵 |
| Australian government | 🖵 | 🖵 | 🖵 |
| Overseas not-for-profit research organisations | 🖵 | 🖵 | 🖵 |
| Overseas universities and research institutes | 🖵 | 🖵 | 🖵 |
| Overseas governments | 🖵 | 🖵 | 🖵 |
| Commercial company | 🖵 | 🖵 | 🖵 |
| Anyone (Publicly available) | 🖵 | 🖵 | 🖵 |

**Q14** What types of research would you share your **anonymous** genomic data with?

|  | Yes | No | Unsure |
| --- | --- | --- | --- |
| Research specific to a condition I have | 🖵 | 🖵 | 🖵 |
| Research into other diseases and conditions | 🖵 | 🖵 | 🖵 |
| General population health research | 🖵 | 🖵 | 🖵 |
| Ancestry research | 🖵 | 🖵 | 🖵 |
| Unspecified future research | 🖵 | 🖵 | 🖵 |

**SECTION C. Your concerns about sharing your genomic data**

**Q15** Consider a scenario where your genomic data is stored as part of your medical record with Queensland Health. How concerned would you be about the following issues if Queensland Health allowed researchers access to your genomics data?

|  | Very concerned | Moderately concerned | Somewhat concerned | Slightly concerned | Not concerned |
| --- | --- | --- | --- | --- | --- |
| Privacy of my personal details (e.g. name, date of birth, address) | 🖵 | 🖵 | 🖵 | 🖵 | 🖵 |
| My genomic data being used for research without my permission | 🖵 | 🖵 | 🖵 | 🖵 | 🖵 |
| My genomic data being used by Queensland Health to improve services or diagnostic tests | 🖵 | 🖵 | 🖵 | 🖵 | 🖵 |
| My genomic data being made publicly available | 🖵 | 🖵 | 🖵 | 🖵 | 🖵 |
| My family finding out about my health results | 🖵 | 🖵 | 🖵 | 🖵 | 🖵 |
| Upsetting my genetic relatives, because my genomic information is similar to theirs | 🖵 | 🖵 | 🖵 | 🖵 | 🖵 |
| Insurance companies using my genomic data to discriminate against me | 🖵 | 🖵 | 🖵 | 🖵 | 🖵 |
| Employers using my genomic data to discriminate against me | 🖵 | 🖵 | 🖵 | 🖵 | 🖵 |
| Ethnic or racial discrimination | 🖵 | 🖵 | 🖵 | 🖵 | 🖵 |
| Being labelled or stigmatised in some way | 🖵 | 🖵 | 🖵 | 🖵 | 🖵 |
| Marketing companies targeting me to sell me products | 🖵 | 🖵 | 🖵 | 🖵 | 🖵 |
| Receiving information about my future health that has no treatment option | 🖵 | 🖵 | 🖵 | 🖵 | 🖵 |
| Police using genomic databases with my details to investigate crimes | 🖵 | 🖵 | 🖵 | 🖵 | 🖵 |

**Q16** Do you have any other concerns about your genomic data from medical records being used for research?

**Option:** [open text box]

- End of survey -
