# Supplementary material for: Sharing genomic data from clinical testing with researchers: public survey of expectations of clinical genomic data management in Queensland, Australia
Source: BMC Med Ethics. 2020 Nov 19;21:119. doi: 10.1186/s12910-020-00563-6 (PMC7678081; doi:10.1186/s12910-020-00563-6)
Supplement: Supplementary file 2 — Additional file 2. Summary tables for statistical analysis of demographic groups. [file 12910_2020_563_MOESM2_ESM.docx]

**Additional File 2**

**Table S1:** Should Queensland Health ask your permission before allowing researchers to access the following from your medical record? – **overall agreement** ^a^

|  | **Identifiable genomic data N (%)** | **Anonymous genomic data N (%)** | **Identifiable biological samples N (%)** | **Anonymous biological samples N (%)** |
| --- | --- | --- | --- | --- |
| **Total (Overall agreement)** | 1281 (86.2) | 530 (35.7) | 1252 (84.4) | 521 (35.3) |
| **Age (years)** | *Total N=1486*  *p=0.001** | *Total N=1483*  *p=0.036* | *Total N=1484*  *p<0.001** | *Total N=1475*  *p=0.032* |
| 18-34 | 55 (94.8) | 29 (50.0) | 55 (94.8) | 29 (50.0) |
| 35-54 | 334 (90.5) | 138 (37.5) | 330 (89.4) | 136 (37.0) |
| 55+ | 891 (84.1) | 362 (34.3) | 867 (82.0) | 355 (33.8) |
| **Gender** | *Total N=1474*  *p=0.014* | *Total N=1471*  *p=0.972* | *Total N=1471*  *p=0.012* | *Total N=1463*  *p=0.821* |
| Male | 403 (83.2) | 172 (35.7) | 393 (81.0) | 167 (34.9) |
| Female | 868 (87.8) | 352 (35.6) | 849 (86.1) | 349 (35.5) |
| **State** | *Total N=1468*  *p=0.326* | *Total N=1465*  *p=0.988* | *Total N=1465*  *p=0.750* | *Total N=1457*  *p=0.684* |
| QLD | 697 (85.3) | 288 (35.5) | 684 (84.0) | 279 (34.6) |
| Non-QLD | 567 (87.1) | 232 (35.5) | 551 (84.6) | 232 (35.6) |
| **Region** | *Total N=1467*  *p=0.735* | *Total N=1464*  *p=0.457* | *Total N=1464*  *p=0.403* | *Total N=1456*  *p=0.627* |
| Metropolitan | 783 (85.9) | 316 (34.7) | 771 (84.9) | 312 (34.6) |
| Regional | 480 (86.5) | 203 (36.6) | 463 (83.3) | 198 (35.8) |
| **ISRAD** | *Total N=1467*  *p=0.190* | *Total N=1464*  *p=0.999* | *Total N=1464*  *p=0.109* | *Total N=1456*  *p=0.578* |
| Low | 276 (83.9) | 117 (35.5) | 268 (81.5) | 111 (33.7) |
| High | 987 (86.7) | 402 (35.5) | 966 (85.1) | 399 (35.4) |
| **Education** | *Total N=1485*  *p<0.001** | *Total N=1482*  *p=0.077* | *Total N=1482*  *p<0.001** | *Total N=1474*  *p=0.136* |
| University | 811 (88.6) | 311 (34.0) | 794 (87.0) | 308 (33.9) |
| Non-university | 468 (82.1) | 219 (38.6) | 456 (80.1) | 213 (37.7) |
| **Worked in life sciences** | *Total N=1480*  *p=0.014* | *Total N=1478*  *p=0.370* | *Total N=1477*  *p=0.026* | *Total N=1470*  *p=0.727* |
| Yes | 97 (94.2) | 41 (39.8) | 94 (92.2) | 38 (36.9) |
| No | 1178 (85.6) | 487 (35.4) | 1153 (83.9) | 481 (35.2) |
| **Worked in health care** | *Total N=1480*  *p=0.002** | *Total N=1477*  *p=0.336* | *Total N=1478*  *P<0.001** | *Total N=1469*  *p=0.615* |
| Yes | 424 (90.2) | 177 (37.5) | 420 (89.7) | 170 (36.3) |
| No | 851 (84.3) | 351 (34.9) | 828 (82.0) | 349 (34.9) |
| **Had genetic or genomic test** | *Total N=1485*  *p=0.688* | *Total N=1482*  *p=0.030* | *Total N=1482*  *p=0.954* | *Total N=1474*  *p=0.027* |
| Yes | 424 (86.7) | 155 (31.8) | 414 (84.5) | 152 (31.3) |
| No | 856 (85.9) | 374 (37.6) | 837 (84.4) | 368 (37.2) |

^a^ *Overall agreement* is *strongly agree* plus *agree*, and is represented as N (%) in this table. It is compared against *overall disagreement* (*strongly disagree*, *disagree* and *unsure*).

* = Statistically significant (p≤0.01).

**Table S2:** How often should Queensland Health ask for permission to give researchers access to the following from your medical records? – **more than once** ^a^

|  | **Identifiable genomic data**  **N (%)** | **Anonymous genomic data**  **N (%)** | **Identifiable biological samples N (%)** | **Anonymous biological samples N (%)** |
| --- | --- | --- | --- | --- |
| **Total (More than once)** | 1019 (68.6) | 380 (25.7) | 1010 (68.2) | 383 (26.0) |
| **Age (years)** | *Total N=1485*  *p<0.001** | *Total N=1480*  *p<0.001** | *Total N=1480*  *p<0.001** | *Total N=1475*  *p<0.001** |
| 18-34 | 48 (82.8) | 19 (32.8) | 48 (82.8) | 21 (36.2) |
| 35-54 | 296 (80.4) | 126 (34.2) | 292 (79.6) | 127 (34.6) |
| 55+ | 674 (63.6) | 234 (22.2) | 669 (63.4) | 234 (22.3) |
| **Gender** | *Total N=1473*  *p<0.001** | *Total N=1468*  *p=0.197* | *Total N=1468*  *p<0.001** | *Total N=1463*  *p=0.339* |
| Male | 293 (60.5) | 113 (23.4) | 291 (60.1) | 116 (24.3) |
| Female | 717 (72.5) | 262 (26.6) | 710 (72.1) | 262 (26.6) |
| **State** | *Total N=1467*  *p=0.003** | *Total N=1462*  *p=0.275* | *Total N=1462*  *p=0.002** | *Total N=1457*  *p=0.233* |
| QLD | 531 (65.1) | 196 (24.3) | 524 (64.6) | 197 (24.4) |
| Non-QLD | 471 (72.4) | 175 (26.8) | 469 (72.0) | 177 (27.2) |
| **Region** | *Total N=1466*  *p=0.054* | *Total N=1461*  *p=0.775* | *Total N=1461*  *p=0.039* | *Total N=1456*  *p=0.982* |
| Metropolitan | 638 (70.1) | 232 (25.6) | 633 (69.9) | 231 (25.6) |
| Regional | 363 (65.3) | 138 (24.9) | 359 (64.7) | 142 (25.6) |
| **ISRAD** | *Total N=1466*  *p=0.690* | *Total N=1461*  *p=0.500* | *Total N=1461*  *p=0.785* | *Total N=1456*  *p=0.354* |
| Low | 221 (67.4) | 88 (26.8) | 220 (67.3) | 91 (27.6) |
| High | 780 (68.5) | 282 (24.9) | 772 (68.1) | 282 (25.0) |
| **Education** | *Total N=1484*  *p<0.001** | *Total N=1479*  *p=0.314* | *Total N=1479*  *p<0.001** | *Total N=1474*  *p=0.229* |
| University | 665 (72.7) | 226 (24.7) | 663 (72.5) | 226 (24.8) |
| Non-university | 352 (61.9) | 153 (27.1) | 345 (61.1) | 156 (27.7) |
| **Worked in life sciences** | *Total N=1479*  *p=0.350* | *Total N=1474*  *p=0.195* | *Total N=1474*  *p=0.434* | *Total N=1470*  *p=0.509* |
| Yes | 75 (72.8) | 21 (20.4) | 74 (71.8) | 24 (23.3) |
| No | 941 (68.4) | 359 (26.2) | 934 (68.1) | 359 (26.3) |
| **Worked in health care** | *Total N=1479*  *p=0.005** | *Total N=1474*  *p=0.456* | *Total N=1474*  *p=0.006** | *Total N=1469*  *p=0.570* |
| Yes | 345 (73.6) | 127 (27.0) | 341 (73.2) | 127 (27.0) |
| No | 670 (66.3) | 253 (25.2) | 665 (66.0) | 256 (25.6) |
| **Had genetic or genomic test** | *Total N=1484*  *p=0.623* | *Total N=1479*  *p=0.112* | *Total N=1479*  *p=0.879* | *Total N=1474*  *p=0.123* |
| Yes | 332 (67.8) | 113 (23.1) | 333 (68.0) | 114 (23.4) |
| No | 686 (69.0) | 266 (26.9) | 676 (68.4) | 268 (27.2) |

^a^ *More than once* is *every time* plus *sometime*, and is represented as N (%) in this table. It is compared against *once or less* (combines *once* and *never*).

* = Statistically significant (p≤0.01).

**Table S3:** Do you think someone else should be able to give permission for researchers to access your anonymous genomic data from medical records if you are no longer able? – **Yes, If so,** Who would you prefer to give permission for your anonymous genomic data to be used in research on your behalf? - **Yes**

|  | **Third party permission**  **N (%)** | **IF Yes (N=538)** | | | | |
| --- | --- | --- | --- | --- | --- | --- |
|  |  | **Family member/**  **Next of kin N (%)** | **Person you legally nominate N (%)** | **Your doctor**  **N (%)** | **Human Research Ethics Committee N (%)** | **Queensland Health Data Governance N (%)** |
| **Total (Yes)** | 642 (43.0) | 392 (61.1) | 294 (45.8) | 125 (19.5) | 121 (18.9) | 61 (9.5) |
| **Age (years)** | *Total N=1490*  *p=0.679* | *Total N=641*  *p=0.005** | *Total N=641*  *p=0.031* | *Total N=641*  *p=0.004** | *Total N=641*  *p=0.384* | *Total N=641*  *p=0.849* |
| 18-34 | 28 (48.3) | 21 (75.0) | 18 (64.3) | 7 (25.0) | 8 (28.6) | 3 (10.7) |
| 35-54 | 155 (42.1) | 109 (70.3) | 79 (51.0) | 16 (10.3) | 30 (19.4) | 13 (8.4) |
| 55+ | 458 (43.1) | 262 (57.2) | 197 (43.0) | 102 (22.3) | 83 (18.1) | 45 (9.8) |
| **Gender** | *Total N=1478*  *p=0.599* | *Total N=636*  *p=0.589* | *Total N=636*  *p=0.819* | *Total N=636*  *p=0.328* | *Total N=636*  *p=0.738* | *Total N=636*  *p=0.094* |
| Male | 204 (42.1) | 122 (59.8) | 92 (45.1) | 44 (21.6) | 36 (17.7) | 25 (12.3) |
| Female | 432 (43.5) | 268 (62.0) | 199 (46.1) | 79 (18.3) | 81 (18.8) | 35 (8.1) |
| **State** | *Total N=1472*  *p=0.061* | *Total N=635*  *p=0.986* | *Total N=635*  *p=0.380* | *Total N=635*  *p=0.158* | *Total N=635*  *p=0.819* | *Total N=635*  *p=0.278* |
| QLD | 371 (45.3) | 226 (60.9) | 164 (44.2) | 80 (21.6) | 69 (18.6) | 39 (10.5) |
| Non-QLD | 264 (40.4) | 161 (61.0) | 126 (47.7) | 45 (17.1) | 51 (19.3) | 21 (8.0) |
| **Region** | *Total N=1471*  *p=0.012* | *Total N=635*  *p=0.520* | *Total N=635 p=0.724* | *Total N=635*  *p=0.952* | *Total N=635*  *p=0.087* | *Total N=635*  *p=0.669* |
| Metropolitan | 418 (45.7) | 251 (60.1) | 193 (46.2) | 82 (19.6) | 87 (20.8) | 38 (9.1) |
| Regional | 217 (39.0) | 136 (62.7) | 97 (44.7) | 43 (19.8) | 33 (15.2) | 22 (10.1) |
| **ISRAD** | *Total N=1471*  *p=0.008** | *Total N=635*  *p=0.718* | *Total N=635*  *p=0.141* | *Total N=635*  *p=0.116* | *Total N=635*  *p=0.770* | *Total N=635*  *p=0.881* |
| Low | 121 (36.8) | 72 (59.5) | 48 (39.7) | 30 (24.8) | 24 (19.8) | 11 (9.1) |
| High | 514 (45.0) | 315 (61.3) | 242 (47.1) | 95 (18.5) | 96 (18.7) | 49 (9.5) |
| **Education** | *Total N=1489*  *p=0.026* | *Total N=641*  *p=0.168* | *Total N=641*  *p=0.007** | *Total N=641*  *p=0.023* | *Total N=641*  *p=0.574* | *Total N=641*  *p=0.886* |
| University | 415 (45.3) | 245 (59.0) | 206 (49.6) | 70 (16.9) | 81 (19.5) | 40 (9.6) |
| Non-university | 226 (39.4) | 146 (64.6) | 87 (38.5) | 55 (24.3) | 40 (17.7) | 21 (9.3) |
| **Worked in life sciences** | *Total N=1484*  *p=0.026* | *Total N=637*  *p=0.323* | *Total N=637*  *p=0.284* | *Total N=637*  *p=0.335* | *Total N=637*  *p=0.006** | *Total N=637*  *p=0.692* |
| Yes | 55 (53.4) | 37 (67.3) | 29 (52.7) | 8 (14.6) | 18 (32.7) | 6 (10.9) |
| No | 582 (42.1) | 352 (60.5) | 263 (45.2) | 116 (19.9) | 102 (17.5) | 54 (9.3) |
| **Worked in health care** | *Total N=1484*  *p=0.476* | *Total N=639*  *p=0.708* | *Total N=639*  *p=0.616* | *Total N=639*  *p=0.054* | *Total N=639*  *p=0.487* | *Total N=639*  *p=0.989* |
| Yes | 210 (44.4) | 126 (60.0) | 93 (44.3) | 32 (15.2) | 43 (20.5) | 20 (9.5) |
| No | 429 (42.4) | 264 (61.5) | 199 (46.4) | 93 (21.7) | 78 (18.2) | 41 (9.6) |
| **Had genetic or genomic test** | *Total N=1489*  *p=0.923* | *Total N=642*  *p=0.856* | *Total N=642*  *p=0.001** | *Total N=642*  *p=0.755* | *Total N=642*  *p=0.541* | *Total N=642*  *p=0.828* |
| Yes | 213 (43.3) | 129 (60.6) | 78 (36.6) | 40 (18.8) | 43 (20.2) | 21 (9.9) |
| No | 429 (43.0) | 263 (61.3) | 216 (50.4) | 85 (19.8) | 78 (18.2) | 40 (9.3) |

^a^ *Yes* response is represented as N (%) in this table.

* = Statistically significant (p≤0.01).

**Table S4:** What organisations would you share your identifiable genomic data with? – **Yes** ^a^

|  | **Australian not-for-profit research organisations**  **N (%)** | **Australian universities and research institutes**  **N (%)** | **Australian government**  **N (%)** | **Overseas not-for-profit research organisations N (%)** | **Overseas universities and research institutes**  **N (%)** | **Overseas governments**  **N (%)** | **Commercial company**  **N (%)** | **Anyone (Publicly available)**  **N (%)** |
| --- | --- | --- | --- | --- | --- | --- | --- | --- |
| **Total (Yes)** | 1076 (72.6) | 1062 (71.6) | 268 (18.4) | 341 (23.4) | 402 (27.5) | 36 (2.5) | 43 (3.0) | 13 (0.9) |
| **Age (years)** | *Total N=1481*  *p=0.002** | *Total N=1484*  *p=0.032* | *Total N=1452*  *p=0.005** | *Total N=1457*  *p=0.002** | *Total N=1461*  *P<0.001** | *Total N=1449*  *p=0.640* | *Total N=1448*  *p=0.163* | *Total N=1449*  *p=0.449* |
| 18-34 | 37 (63.8) | 43 (74.1) | 16 (27.6) | 15 (25.9) | 21 (36.2) | 2 (3.5) | 4 (6.9) | 0 (0) |
| 35-54 | 245 (66.6) | 243 (66.2) | 49 (13.4) | 60 (16.5) | 69 (18.9) | 11 (3.0) | 12 (3.3) | 5 (1.4) |
| 55+ | 794 (75.3) | 776 (73.3) | 203 (19.8) | 266 (25.7) | 312 (30.1) | 23 (2.2) | 27 (2.6) | 8 (0.8) |
| **Gender** | *Total N=1469*  *p=0.066* | *Total N=1471*  *p=0.298* | *Total N=1440*  *p<0.001** | *Total N=1445*  *p=0.083* | *Total N=1449*  p=0.152 | *Total N=1437*  p=0.254 | *Total N=1436*  p=0.055 | *Total N=1437*  p=0.104 |
| Male | 366 (75.6) | 355 (73.2) | 126 (26.5) | 124 (26.0) | 142 (29.7) | 15 (3.2) | 20 (4.2) | 7 (1.5) |
| Female | 700 (71.1) | 696 (70.6) | 140 (14.5) | 212 (21.9) | 253 (26.1) | 21 (2.2) | 23 (2.4) | 6 (0.6) |
| **State** | *Total N=1464*  *p=0.060* | *Total N=1465*  *p=0.004** | *Total N=1434*  *p=0.008** | *Total N=1439*  *p=0.736* | *Total N=1443*  *p=0.252* | *Total N=1431*  *p=0.581* | *Total N=1430*  *p=0.196* | *Total N=1431*  *p=0.652* |
| QLD | 609 (74.9) | 611 (75.0) | 166 (20.9) | 186 (23.2) | 232 (28.9) | 21 (2.7) | 28 (3.5) | 8 (1.0) |
| Non-QLD | 459 (70.5) | 443 (68.2) | 98 (15.4) | 153 (24.0) | 168 (26.2) | 14 (2.2) | 15 (2.4) | 5 (0.8) |
| **Region** | *Total N=1463*  *p=0.053* | *Total N=1464*  *p=0.267* | *Total N=1433*  *p=0.010** | *Total N=1438*  *p=0.583* | *Total N=1442*  *p=0.128* | *Total N=1430*  *p=0.541* | *Total N=1429*  *p=0.929* | *Total N=1430*  *p=0.238* |
| Metropolitan | 647 (71.2) | 646 (70.9) | 182 (20.5) | 206 (23.1) | 236 (26.3) | 20 (2.3) | 27 (3.0) | 6 (0.7) |
| Regional | 420 (75.8) | 407 (73.6) | 82 (15.1) | 133 (24.4) | 164 (30.0) | 15 (2.8) | 16 (3.0) | 7 (1.3) |
| **ISRAD** | *Total N=1463*  *p=0.089* | *Total N=1464*  *p=0.759* | *Total N=1433*  *p=0.114* | *Total N=1438*  *p=0.837* | *Total N=1442*  *p=0.765* | *Total N=1430*  *p=0.969* | *Total N=1429*  *p=0.917* | *Total N=1430*  *p=0.487* |
| Low | 252 (76.6) | 233 (71.3) | 50 (15.4) | 78 (24.0) | 92 (28.4) | 8 (2.5) | 10 (3.1) | 4 (1.2) |
| High | 815 (71.9) | 820 (72.1) | 214 (19.3) | 261 (23.5) | 308 (27.6) | 27 (2.4) | 33 (3.0) | 9 (0.8) |
| **Education** | *Total N=1480*  *p<0.001** | *Total N=1482*  *p<0.001** | *Total N=1451*  *p=0.111* | *Total N=1456*  *p=0.226* | *Total N=1460*  *p=0.323* | *Total N=1448*  *p=0.255* | *Total N=1447*  *p=0.381* | *Total N=1448*  *p=0.542* |
| University | 624 (68.4) | 624 (68.4) | 155 (17.2) | 202 (22.4) | 241 (26.6) | 19 (2.1) | 24 (2.7) | 7 (0.8) |
| Non-university | 452 (79.7) | 438 (77.0) | 113 (20.6) | 139 (25.1) | 161 (29.0) | 17 (3.1) | 19 (3.5) | 6 (1.1) |
| **Worked in life sciences** | *Total N=1475*  *p=0.124* | *Total N=1477*  *p=0.485* | *Total N=1446*  *p=0.245* | *Total N=1451*  *p=0.094* | *Total N=1455*  *p=0.082* | *Total N=1443*  *p=0.019* | *Total N=1442*  *p=0.064* | *Total N=1443*  *p=0.930* |
| Yes | 68 (66.0) | 70 (68.6) | 23 (22.6) | 31 (30.1) | 36 (35.0) | 6 (5.9) | 6 (5.9) | 1 (1.0) |
| No | 1002 (73.0) | 988 (71.9) | 241 (17.9) | 308 (22.9) | 365 (27.0) | 29 (2.2) | 36 (2.7) | 12 (1.0) |
| **Worked in health care** | *Total N=1475*  *p=0.001** | *Total N=1477*  *p=0.040* | *Total N=1446*  *p=0.009** | *Total N=1451*  *p=0.105* | *Total N=1455*  *p=0.084* | *Total N=1443*  *p=0.043* | *Total N=1442*  *p=0.648* | *Total N=1444*  *p=0.604* |
| Yes | 315 (67.0) | 318 (68.0) | 67 (14.6) | 96 (20.7) | 113 (24.5) | 17 (3.7) | 15 (3.3) | 5 (1.1) |
| No | 755 (75.1) | 738 (73.1) | 201 (20.3) | 243 (24.6) | 286 (28.8) | 19 (1.9) | 28 (2.8) | 8 (0.8) |
| **Had genetic or genomic test** | *Total N=1480*  p=0.050 | *Total N=1482*  p=0.030 | *Total N=1451*  p=0.122 | *Total N=1456*  p=0.034 | *Total N=1460*  *p=0.026* | *Total N=1448*  *p=0.729* | *Total N=1447*  *p=0.721* | *Total N=1448*  *p=0.834* |
| Yes | 371 (75.9) | 370 (75.2) | 100 (20.6) | 130 (26.8) | 152 (32.2) | 13 (2.7) | 13 (2.7) | 4 (0.8) |
| No | 704 (71.0) | 691 (69.8) | 167 (17.3) | 211 (21.8) | 250 (25.7) | 23 (2.4) | 29 (3.0) | 9 (0.9) |

^a^ *Yes* is represented as N (%) in this table. It is compared against *no* (combines *no* and *unsure*).

* = Statistically significant (p≤0.01).

**Table S5:** What organisations would you share your anonymous genomic data with? - **Yes** ^a^

|  | **Australian not-for-profit research organisations**  **N (%)** | **Australian universities and research institutes**  **N (%)** | **Australian government**  **N (%)** | **Overseas not-for-profit research organisations**  **N (%)** | **Overseas universities and research institutes**  **N (%)** | **Overseas governments**  **N (%)** | **Commercial company**  **N (%)** | **Anyone (Publicly available)**  **N (%)** |
| --- | --- | --- | --- | --- | --- | --- | --- | --- |
| **Total (Yes)** | 1361 (91.9) | 1359 (91.8) | 720 (49.6) | 797 (54.5) | 842 (57.5) | 247 (17.1) | 247 (17.1) | 187 (13.0) |
| **Age (years)** | *Total N=1480*  *p=0.025* | *Total N=1479*  *p=0.082* | *Total N=1452*  *p=0.496* | *Total N=1461*  *p=0.029* | *Total N=1464*  *p=0.039* | *Total N=1448*  *p=0.015* | *Total N=1447*  *p=0.149* | *Total N=1442*  *p=0.011* |
| 18-34 | 55 (94.8) | 56 (96.6) | 31 (53.5) | 36 (62.1) | 39 (67.2) | 18 (31.0) | 15 (25.9) | 15 (25.9) |
| 35-54 | 327 (88.6) | 330 (89.4) | 173 (47.1) | 179 (48.9) | 193 (52.6) | 62 (17.0) | 65 (17.9) | 47 (13.0) |
| 55+ | 978 (92.9) | 972 (92.4) | 516 (50.2) | 582 (56.1) | 610 (58.7) | 167 (16.3) | 167 (16.3) | 125 (12.2) |
| **Gender** | *Total N=1468*  *p=0.661* | *Total N=1467*  *p=0.555* | *Total N=1440*  *p=0.002** | *Total N=1449*  *p=0.082* | *Total N=1452*  *p=0.684* | *Total N=1436*  *p=0.077* | *Total N=1435*  *p=0.018* | *Total N=1430*  *p=0.009** |
| Male | 446 (92.3) | 440 (91.3) | 262 (55.3) | 273 (57.7) | 277 (58.2) | 92 (19.6) | 97 (20.4) | 77 (16.3) |
| Female | 903 (91.7) | 908 (92.2) | 452 (46.8) | 516 (52.9) | 557 (57.1) | 153 (15.8) | 148 (15.4) | 109 (11.4) |
| **State** | *Total N=1462*  *p=0.184* | *Total N=1461*  *p=0.016* | *Total N=1434*  *p=0.061* | *Total N=1444*  *p=0.843* | *Total N=1446*  *p=0.475* | *Total N=1430*  *p=0.352* | *Total N=1429*  *p=0.240* | *Total N=1424*  *p=0.181* |
| QLD | 754 (93.0) | 759 (93.6) | 410 (52.0) | 439 (55.2) | 468 (58.7) | 142 (18.0) | 144 (18.3) | 111 (14.1) |
| Non-QLD | 593 (91.1) | 586 (90.2) | 304 (47.1) | 354 (54.6) | 369 (56.9) | 104 (16.2) | 102 (15.9) | 75 (11.7) |
| **Region** | *Total N=1461*  *p=0.280* | *Total N=1460*  *p=0.104* | *Total N=1433*  *p=0.038* | *Total N=1443*  *p=0.069* | *Total N=1445*  *p=0.442* | *Total N=1429*  *p=0.029* | *Total N=1428*  *p=0.101* | *Total N=1423*  *p=0.058* |
| Metropolitan | 841 (92.7) | 844 (93.0) | 461 (52.0) | 508 (56.8) | 526 (58.7) | 167 (18.9) | 164 (18.5) | 127 (14.4) |
| Regional | 505 (91.2) | 500 (91.0) | 253 (46.3) | 285 (51.9) | 311 (56.7) | 79 (14.4) | 82 (15.1) | 59 (10.9) |
| **ISRAD** | *Total N=1461*  *p=0.470* | *Total N=1460*  *p=0.060* | *Total N=1433*  *p=0.032* | *Total N=1443*  *p=0.158* | *Total N=1445*  *p=0.238* | *Total N=1429*  *p=0.013* | *Total N=1428*  *p=0.389* | *Total N=1423*  *p=0.009** |
| Low | 300 (91.9) | 292 (90.0) | 144 (44.6) | 168 (51.5) | 179 (55.1) | 41 (12.7) | 50 (15.6) | 28 (8.8) |
| High | 1046 (92.4) | 1052 (92.8) | 570 (51.4) | 625 (56.0) | 658 (58.8) | 205 (18.6) | 196 (17.7) | 158 (14.3) |
| **Education** | *Total N=1479*  *p=0.961* | *Total N=1478*  *p=0.495* | *Total N=1451*  *p=0.036* | *Total N=1460*  *p=0.141* | *Total N=1463*  *p=0.053* | *Total N=1447*  *p=0.780* | *Total N=1446*  *p=0.958* | *Total N=1441*  *p=0.125* |
| University | 835 (91.9) | 839 (92.2) | 465 (51.8) | 506 (56.1) | 538 (59.5) | 151 (16.9) | 152 (17.0) | 125 (14.0) |
| Non-university | 524 (91.9) | 518 (91.2) | 255 (46.1) | 291 (52.2) | 303 (54.3) | 96 (17.4) | 95 (17.2) | 62 (11.3) |
| **Worked in life sciences** | *Total N=1474*  *p=0.036* | *Total N=1473*  *p=0.330* | *Total N=1446*  *p=0.079* | *Total N=1455*  *p=0.146* | *Total N=1458*  *p=0.225* | *Total N=1442*  *p=0.648* | *Total N=1441*  *p=0.857* | *Total N=1436*  *p=0.785* |
| Yes | 89 (86.4) | 92 (89.3) | 59 (57.8) | 62 (61.4) | 65 (63.1) | 19 (18.6) | 18 (17.7) | 12 (12.0) |
| No | 1265 (92.3) | 1261 (92.0) | 656 (48.8) | 730 (53.9) | 772 (57.0) | 226 (16.9) | 227 (17.0) | 173 (13.0) |
| **Worked in health care** | *Total N=1474*  *p=0.009** | *Total N=1473*  *p=0.030* | *Total N=1447*  *p=0.001** | *Total N=1455*  *p=0.012* | *Total N=1458*  *p=0.099* | *Total N=1444*  *p=0.176* | *Total N=1442*  *p=0.808* | *Total N=1437*  *p=0.309* |
| Yes | 418 (89.1) | 418 (89.5) | 200 (43.5) | 230 (49.7) | 253 (54.3) | 70 (15.2) | 77 (16.8) | 53 (11.6) |
| No | 936 (93.1) | 934 (92.8) | 519 (52.6) | 563 (56.8) | 584 (58.9) | 177 (18.0) | 170 (17.3) | 133 (13.6) |
| **Had genetic or genomic test** | *Total N=1479*  *p=0.918* | *Total N=1478*  *p=0.170* | *Total N=1451*  *p=0.025* | *Total N=1460*  *p=0.010** | *Total N=1463*  *p=0.026* | *Total N=1447*  *p=0.791* | *Total N=1446*  *p=0.349* | *Total N=1441*  *p=0.643* |
| Yes | 452 (92.1) | 457 (93.3) | 262 (53.7) | 288 (59.3) | 301 (61.6) | 81 (16.6) | 89 (18.3) | 65 (13.5) |
| No | 908 (91.9) | 901 (91.2) | 457 (47.5) | 508 (52.2) | 540 (55.4) | 165 (17.2) | 157 (16.4) | 121 (12.6) |

^a^ *Yes* is represented as N (%) in this table. It is compared against *no* (combines *no* and *unsure*).

* = Statistically significant (p≤0.01).

**Table S6:** What types of research would you share your anonymous genomic data with? – **Yes** ^a^

|  | **Research specific to a condition I have**  **N (%)** | **Research into other diseases and conditions**  **N (%)** | **General population health research**  **N (%)** | **Ancestry research**  **N (%)** | **Unspecified future research**  **N (%)** |
| --- | --- | --- | --- | --- | --- |
| **Total (Yes)** | 1,407 (95.1) | 1,341 (90.6) | 1,285 (86.7) | 961 (65.5) | 717 (48.9) |
| **Age (years)** | *Total N=1479*  p=0.871 | *Total N=1480*  p=0.445 | *Total N=1482*  p=0.280 | *Total N=1466*  p=0.010* | *Total N=1466*  p=0.138 |
| 18-34 | 54 (94.7) | 52 (91.2) | 49 (86.0) | 37 (64.9) | 29 (50.9) |
| 35-54 | 349 (94.6) | 327 (88.9) | 310 (84.2) | 217 (59.1) | 163 (44.4) |
| 55+ | 1003 (95.3) | 961 (91.1) | 925 (87.5) | 707 (67.9) | 525 (50.4) |
| **Gender** | *Total N=1467*  *p=0.205* | *Total N=1468*  *p=0.174* | *Total N=1470*  *p=0.332* | *Total N=1454 p=0.933* | *Total N=1454*  *p=0.267* |
| Male | 454 (94.0) | 431 (89.1) | 413 (85.5) | 315 (65.8) | 244 (51.1) |
| Female | 940 (95.5) | 898 (91.3) | 862 (87.3) | 639 (65.5) | 468 (48.0) |
| **State** | *Total N=1462*  *p=0.040* | *Total N=1463*  *p=0.006** | *Total N=1465*  *p=0.625* | *Total N=1450*  *p=0.004** | *Total N=1450*  *p=0.029* |
| QLD | 781 (96.3) | 755 (92.8) | 711 (87.4) | 556 (69.2) | 415 (51.7) |
| Non-QLD | 612 (94.0) | 575 (88.6) | 563 (86.5) | 400 (61.9) | 297 (45.9) |
| **Region** | *Total N=1461*  *p=0.632* | *Total N=1462*  *p=0.751* | *Total N=1464*  *p=0.217* | *Total N=1449*  *p=0.773* | *Total N=1449*  *p=0.766* |
| Metropolitan | 867 (95.5) | 828 (91.1) | 799 (87.8) | 595 (66.3) | 444 (49.4) |
| Regional | 525 (95.0) | 501 (90.6) | 474 (85.6) | 361 (65.5) | 268 (48.6) |
| **ISRAD** | *Total N=1461*  *p=0.896* | *Total N=1462*  *p=0.800* | *Total N=1464*  *p=0.087* | *Total N=1449*  *p=0.029* | *Total N=1449*  *p=0.074* |
| Low | 312 (95.4) | 297 (90.6) | 276 (84.2) | 198 (60.9) | 146 (44.8) |
| High | 1080 (95.2) | 1032 (91.0) | 997 (87.8) | 758 (67.4) | 566 (50.4) |
| **Education** | *Total N=1478*  *p=0.604* | *Total N=1474*  *p=0.721* | *Total N=1481*  *p=0.221* | *Total N=1465*  *p<0.001* | *Total N=1465*  *p=0.006** |
| University | 862 (94.8) | 821 (90.3) | 797 (87.5) | 557 (61.7) | 416 (46.0) |
| Non-university | 543 (95.4) | 518 (90.9) | 486 (85.3) | 403 (71.7) | 300 (53.5) |
| **Worked in life sciences** | *Total N=1473*  *p=0.062* | *Total N=1474*  *p=0.231* | *Total N=1476*  *p=0.586* | *Total N=1460*  *p=0.007** | *Total N=1460*  *p=0.739* |
| Yes | 93 (91.2) | 88 (87.1) | 91 (88.4) | 55 (53.4) | 52 (50.5) |
| No | 1307 (95.3) | 1246 (90.8) | 1187 (86.5) | 902 (66.5) | 662 (48.8) |
| **Worked in health care** | *Total N=1473*  *p=0.138* | *Total N=1474*  *p=0.042* | *Total N=1476*  *p=0.192* | *Total N=1460*  *p<0.001** | *Total N=1460*  *p<0.001** |
| Yes | 440 (93.8) | 412 (88.2) | 399 (84.9) | 273 (58.8) | 198 (42.7) |
| No | 960 (95.6) | 922 (91.6) | 879 (87.4) | 683 (68.6) | 515 (51.7) |
| **Had genetic or genomic test** | *Total N=1478*  *p=0.326* | *Total N=1479*  *p=0.036* | *Total N=1481*  *p=0.013* | *Total N=1465*  *p<0.001** | *Total N=1465*  *p=0.096* |
| Yes | 469 (95.9) | 455 (92.9) | 441 (89.8) | 357 (73.0) | 253 (52.0) |
| No | 937 (94.7) | 885 (89.5) | 843 (85.2) | 603 (61.8) | 463 (47.3) |

^a^ *Yes* is represented as N (%) in this table. It is compared against *no* (combines *no* and *unsure*).

* = Statistically significant (p≤0.01).

**Table S7a:** Consider a scenario where your genomic data is stored as part of your medical record with Queensland Health. How concerned would you be about the following issues if Queensland Health allowed researchers access to your genomics data? – **Very or moderately concerned** ^a^

|  | **Privacy of my personal details (e.g. name, date of birth, address)**  **N (%)** | **My genomic data being used for research without my permission**  **N (%)** | **My genomic data being used by Queensland Health to improve services or diagnostic tests**  **N (%)** | **My genomic data being made publicly available**  **N (%)** | **My family finding out about my health results**  **N (%)** | **Upsetting my genetic relatives, because my genomic information is similar to theirs**  **N (%)** | **Insurance companies using my genomic data to discriminate against me**  **N (%)** | **Employers using my genomic data to discriminate against me**  **N (%)** |
| --- | --- | --- | --- | --- | --- | --- | --- | --- |
| **Total (Very or moderately concerned )** | 1109 (76.1) | 943 (64.8) | 503 (34.6) | 1179 (80.9) | 460 (31.7) | 456 (31.4) | 1307 (89.9) | 1266 (87.0) |
| **Age (years)** | *Total N=1456 p=0.274* | *Total N=1454 p=0.069* | *Total N=1451*  *p=0.499* | *Total N=1456 p=0.095* | *Total N=1452 p=0.191* | *Total N=1453 p=0.166* | *Total N=1453 p=0.203* | *Total N=1454 p=0.332* |
| 18-34 | 41 (74.6) | 34 (61.8) | 16 (29.1) | 43 (78.2) | 18 (32.7) | 15 (27.3) | 47 (85.5) | 45 (81.8) |
| 35-54 | 286 (79.2) | 252 (69.8) | 132 (36.6) | 306 (84.8) | 128 (35.5) | 127 (35.3) | 332 (92.0) | 319 (88.6) |
| 55+ | 781 (75.1) | 656 (63.2) | 355 (34.3) | 829 (79.7) | 314 (30.3) | 314 (30.3) | 927 (89.4) | 901 (86.7) |
| **Gender** | *Total N=1445 p=0.069* | *Total N=1443*  *p=0.003** | *Total N=1440 p=0.185* | *Total N=1445 p=0.148* | *Total N=1441 p=0.259* | *Total N=1442 p=0.995* | *Total N=1442 p=0.984* | *Total N=1443 p=0.589* |
| Male | 348 (73.4) | 282 (59.6) | 153 (32.4) | 374 (78.9) | 140 (29.6) | 148 (31.4) | 425 (89.9) | 409 (86.3) |
| Female | 755 (77.8) | 655 (67.5) | 347 (35.9) | 797 (82.2) | 315 (32.5) | 304 (31.3) | 871 (89.9) | 846 (87.3) |
| **State** | *Total N=1439 p<0.001** | *Total N=1437*  *p=0.007** | *Total N=1434 p=0.001** | *Total N=1439*  *p=0.189* | *Total N=1435 p=0.003** | *Total N=1436 p=0.008** | *Total N=1436 p<0.001** | *Total N=1437 p=0.001** |
| QLD | 575 (72.0) | 491 (61.6) | 243 (30.6) | 636 (79.6) | 225 (28.3) | 225 (28.2) | 696 (87.3) | 673 (84.3) |
| Non-QLD | 517 (80.8) | 438 (68.4) | 250 (39.1) | 527 (82.3) | 227 (35.5) | 221 (34.7) | 594 (92.3) | 577 (90.3) |
| **Region** | *Total N=1438 p=0.310* | *Total N=1436 p=0.334* | *Total N=1433 p=0.833* | *Total N=1438 p=0.764* | *Total N=1434 p=0.566* | *Total N=1435 p=0.412* | *Total N=1435 p=0.921* | *Total N=1436 p=0.703* |
| Metropolitan | 668 (75.0) | 566 (63.7) | 307 (34.6) | 717 (80.6) | 275 (31.0) | 269 (30.3) | 798 (89.8) | 770 (86.7) |
| Regional | 423 (77.3) | 362 (66.2) | 186 (34.1) | 445 (81.2) | 177 (32.4) | 177 (32.4) | 491 (89.9) | 479 (87.4) |
| **ISRAD** | *Total N=1438 p=0.922* | *Total N=1436 p=0.825* | *Total N=1433 p=0.578* | *Total N=1438 p=0.659* | *Total N=1434 p=0.283* | *Total N=1435 p=0.255* | *Total N=1435 p=0.273* | *Total N=1436 p=0.297* |
| Low | 248 (76.1) | 209 (64.1) | 116 (35.7) | 267 (81.7) | 111 (33.9) | 110 (33.6) | 299 (91.4) | 290 (88.7) |
| High | 843 (75.8) | 719 (64.8) | 377 (34.0) | 895 (80.6) | 341 (30.8) | 336 (30.3) | 990 (89.4) | 959 (86.5) |
| **Education** | *Total N=1455 p=0.215* | *Total N=1453 p=0.679* | *Total N=1450*  *p=0.555* | *Total N=1455 p=0.021* | *Total N=1451 p=0.450* | *Total N=1452 p=0.149* | *Total N=1452 p=0.029* | *Total N=1453 p=0.730* |
| University | 690 (77.2) | 582 (65.2) | 303 (343.0) | 740 (82.8) | 289 (32.4) | 291 (32.7) | 813 (91.3) | 779 (87.2) |
| Non-university | 417 (74.3) | 359 (64.1) | 198 (35.5) | 437 (77.9) | 170 (30.5) | 163 (29.1) | 492 (87.7) | 485 (86.6) |
| **Worked in life sciences** | *Total N=1450 p=0.456* | *Total N=1448 p=0.651* | *Total N=1445 p=0.772* | *Total N=1450 p=0.591* | *Total N=1447 p=0.601* | *Total N=1448 p=0.554* | *Total N=1447 p=0.459* | *Total N=1448 p=0.746* |
| Yes | 73 (73.0) | 67 (67.0) | 36 (36.0) | 83 (83.0) | 34 (34.0) | 34 (34.0) | 92 (92.0) | 88 (88.0) |
| No | 1030 (76.3) | 873 (64.8) | 465 (34.6) | 1091 (80.8) | 424 (31.5) | 420 (31.2) | 1208 (89.7) | 1171 (86.9) |
| **Worked in health care** | *Total N=1450 p=0.561* | *Total N=1448 p=0.057* | *Total N=1445 p=0.162* | *Total N=1450 p=0.634* | *Total N=1446 p=0.002** | *Total N=1447 p=0.004** | *Total N=1447*  *p=0.487* | *Total N=1448 p=0.587* |
| Yes | 358 (77.2) | 317 (68.3) | 172 (37.2) | 379 (81.7) | 171 (36.9) | 169 (36.4) | 420 (90.7) | 407 (87.7) |
| No | 747 (75.8) | 622 (63.2) | 328 (33.4) | 795 (80.6) | 285 (29.0) | 284 (28.9) | 881 (89.5) | 853 (86.7) |
| **Had genetic or genomic test** | *Total N=1455 p=0.133* | *Total N=1453 p=0.224* | *Total N=1450 p=0.002** | *Total N=1455 p=0.115* | *Total N=1451 p=0.007** | *Total N=1452 p=0.001** | *Total N=1452 p=0.151* | *Total N=1453 p=0.262* |
| Yes | 359 (73.7) | 304 (62.7) | 142 (29.3) | 382 (78.6) | 131 (27.0) | 125 (25.7) | 429 (88.3) | 416 (85.6) |
| No | 748 (77.3) | 638 (65.9) | 360 (37.3) | 795 (82.0) | 328 (34.0) | 330 (34.2) | 876 (90.7) | 848 (87.7) |

^a^ *Very or moderately concerned* is combined and represented as N (%) in this table. It is compared against *lower concern* (combines *somewhat*, *slightly* and *not concerned*).

* = Statistically significant (p≤0.01)

**Table S7b:** Consider a scenario where your genomic data is stored as part of your medical record with Queensland Health. How concerned would you be about the following issues if Queensland Health allowed researchers access to your genomics data? – **Very or moderately concerned**

|  | **Ethnic or racial discrimination**  **N (%)** | **Being labelled or stigmatised in some way**  **N (%)** | **Marketing companies targeting me to sell me products N (%)** | **Receiving information about my future health that has no treatment option N (%)** | **Police using genomic databases with my details to investigate crimes N (%)** |
| --- | --- | --- | --- | --- | --- |
| **Total (Very or moderately concerned )** | 1019 (70.4) | 1068 (73.5) | 1323 (90.9) | 753 (51.8) | 716 (49.3) |
| **Age (years)** | *Total N=1446 p<0.001** | *Total N=1453 p=0.137* | *Total N=1454*  *p<0.001** | *Total N=1454*  *p=0.075* | *Total N=1452*  *p=0.295* |
| 18-34 | 29 (52.7) | 36 (65.5) | 44 (80.0) | 20 (37.0) | 21 (38.9) |
| 35-54 | 236 (65.6) | 255 (70.8) | 317 (87.8) | 193 (53.6) | 179 (49.6) |
| 55+ | 754 (73.1) | 776 (74.8) | 961 (92.6) | 540 (51.9) | 516 (49.8) |
| **Gender** | *Total N=1435 p=0.522* | *Total N=1442 p=0.423* | *Total N=1443*  *p=0.357* | *Total N=1443*  *p=0.033* | *Total N=1441*  *p=0.699* |
| Male | 336 (71.5) | 340 (72.0) | 436 (92.0) | 226 (47.7) | 236 (50.0) |
| Female | 674 (69.8) | 718 (74.0) | 877 (90.5) | 520 (53.7) | 474 (48.9) |
| **State** | *Total N=1429 p<0.001** | *Total N=1436 p<0.001** | *Total N=1437 p=0.006** | *Total N=1437*  *p=0.122* | *Total N=1435 p<0.001** |
| QLD | 526 (66.3) | 558 (70.0) | 711 (89.1) | 398 (49.8) | 355 (44.5) |
| Non-QLD | 480 (75.5) | 498 (77.9) | 596 (93.3) | 344 (53.9) | 350 (54.9) |
| **Region** | *Total N=1428 p=0.844* | *Total N=1435*  *p=0.928* | *Total N=1436*  *p=0.217* | *Total N=1436*  *p=0.774* | *Total N=1434*  *p=0.824* |
| Metropolitan | 623 (70.6) | 652 (73.5) | 802 (90.2) | 462 (52.0) | 438 (49.3) |
| Regional | 383 (70.2) | 404 (73.7) | 504 (92.1) | 280 (51.2) | 266 (48.7) |
| **ISRAD** | *Total N=1428*  *p=0.104* | *Total N=1435 p=0.078* | *Total N=1436*  *p=0.452* | *Total N=1436*  *p=0.088* | *Total N=1434*  *p=0.287* |
| Low | 240 (74.1) | 253 (77.4) | 299 (92.0) | 182 (55.8) | 168 (51.7) |
| High | 766 (69.4) | 803 (72.5) | 1007 (90.6) | 560 (50.5) | 536 (48.3) |
| **Education** | *Total N=1445*  *p=0.785* | *Total N=1452 p=0.378* | *Total N=1453*  *p=0.352* | *Total N=1453*  *p=0.172* | *Total N=1451*  *p=0.866* |
| University | 624 (70.2) | 649 (72.7) | 806 (90.4) | 449 (50.3) | 440 (49.4) |
| Non-university | 394 (70.9) | 418 (74.8) | 515 (91.8) | 303 (54.0) | 274 (48.9) |
| **Worked in life sciences** | *Total N=1440*  *p=0.696* | *Total N=1448 p=0.741* | *Total N=1448*  *p=0.162* | *Total N=1448*  *p=0.163* | *Total N=1446*  *p=0.786* |
| Yes | 68 (68.7) | 72 (72.0) | 87 (87.0) | 45 (45.0) | 48 (48.0) |
| No | 946 (70.5) | 991 (73.5) | 1229 (91.2) | 704 (52.2) | 665 (49.4) |
| **Worked in health care** | *Total N=1440 p=0.936* | *Total N=1447 p=0.853* | *Total N=1448*  *p=0.419* | *Total N=1448*  *p=0.967* | *Total N=1446*  *p=0.028* |
| Yes | 327 (70.6) | 342 (73.7) | 417 (90.1) | 239 (51.5) | 247 (53.5) |
| No | 688 (70.4) | 720 (73.3) | 900 (91.4) | 508 (51.6) | 465 (47.3) |
| **Had genetic or genomic test** | *Total N=1445 p=0.002** | *Total N=1452 p=0.002** | *Total N=1453*  *p=0.129* | *Total N=1453*  *p<0.001** | *Total N=1451*  *p=0.001** |
| Yes | 315 (65.1) | 333 (68.5) | 434 (89.3) | 218 (45.0) | 208 (42.9) |
| No | 703 (73.2) | 734 (76.0) | 887 (91.7) | 534 (55.2) | 507 (52.5) |

^a^ *Very or moderately concerned* is combined and represented as N (%) in this table. It is compared against *lower concern* (combines *somewhat*, *slightly* and *not concerned*).

* = Statistically significant (p≤0.01)
